# Living, Heat-Killed *Limosilactobacillus mucosae* and Its Cell-Free Supernatant Differentially Regulate Colonic Serotonin Receptors and Immune Response in Experimental Colitis

**DOI:** 10.3390/nu16040468

**Published:** 2024-02-06

**Authors:** Zhiyuan Sun, Siqi Huang, Xing Yan, Xiuwen Zhang, Youling Hao, Lili Jiang, Zhaolai Dai

**Affiliations:** 1 State Key Laboratory of Animal Nutrition and Feeding, China Agricultural University, Beijing 100193, China; hrszy@126.com (Z.S.); yan_xing0314@163.com (X.Y.); sammierr@163.com (X.Z.); hao17863527642@163.com (Y.H.); lilijiang2020@163.com (L.J.); 2 College of Animal Science and Technology, China Agricultural University, Beijing 100193, China; litdip-hsq@163.com

**Keywords:** *Limosilactobacillus mucosae*, heat killed, supernatant, serotonin, indole-3-acetic acid

## Abstract

*Lactobacillus* species have been shown to alleviate gut inflammation and oxidative stress. However, the effect of different lactobacilli components on gut inflammation has not been well studied. This study aims to identify the differences in the effect and mechanisms of different forms and components of *Limosilactobacillus mucosae* (LM) treatment in the alleviation of gut inflammation using a colitis mouse model that is induced by dextran sodium sulfate (DSS). Seventy-two C57BL/6 mice were divided into six groups: control, DSS, live LM+DSS (LM+DSS), heat-killed LM+DSS (HKLM+DSS), LM cell-free supernatant + DSS (LMCS+DSS), and MRS medium + DSS (MRS+DSS). The mice were treated with different forms and components of LM for two weeks before DSS treatment. After that, the mice were sacrificed for an assessment of their levels of inflammatory cytokines, serotonin (5-HT) receptors (HTRs), and tryptophan metabolites. The results showed that, compared to other treatments, LMCS was more effective (*p* < 0.05) in the alleviation of DSS-induced body weight loss and led to an increase in the disease activity index score. All three forms and components of LM increased (*p* < 0.05) the levels of indole-3-acetic acid but reduced (*p* < 0.05) the levels of 5-HT in the colon. HKLM or LMCS reduced (*p* < 0.05) the percentages of CD3^+^CD8^+^ cytotoxic T cells but increased (*p* < 0.05) the percentages of CD3^+^CD4^+^ T helper cells in the spleen. LM or HKLM increased (*p* < 0.05) abundances of CD4^+^Foxp3^+^ regulatory T cells in the spleen. The LM and LMCS treatments reduced (*p* < 0.05) the expression of the pro-inflammatory cytokines *Il6* and *Il17a*. The mice in the HKLM+DSS group had higher (*p* < 0.05) mRNA levels of the anti-inflammatory cytokine *Il10*, the cell differentiation and proliferation markers *Lgr5* and *Ki67*, the 5-HT degradation enzyme *Maoa*, and HTRs (*Htr1a*, *Htr2a*, and *Htr2b*) in the colon. All three forms and components of LM reduced the phosphorylation of STAT3. The above findings can help to optimize the functionality of probiotics and develop new dietary strategies that aid in the maintenance of a healthy gut.

## 1. Introduction

The functional robustness of the digestive tract is crucial for maintaining the nutrition and immunity of the body in both humans and animals [1]. However, dietary factors, infection, or weaning can induce oxidative stress and inflammation in the gut, which have been proven to damage the gut barrier [2,3,4]. The gut barrier is a critical host–environment interaction interface that is regulated by biochemical factors in the gut lumen [5]. The gut immune system plays a key role in this perturbation of homeostasis between commensal bacteria and the function of the gut barrier [6]. Its response involves the activation of a variety of intestinal immune cells, including neutrophils, macrophages, T cells, B cells, and dendritic cells [7,8]. It has been observed that the pro-inflammatory CD8^+^ cytotoxic T lymphocytes can induce a relapse of colitis via the production of interferon-γ in mice [9]. These are paralleled by the production of inflammatory cytokines such as tumor necrosis factor-α (TNF-α), interleukin-1β (IL-1β), IL-17A, and IL-6, as well as chemokines [8]. In addition, the anti-inflammatory regulatory T (Treg) cells, together with T helper cells, play an important role in modulating gut immune homeostasis [10]. Treg cells have been found to be reduced in an inflamed colon with increased levels of IL-17 [11,12,13,14]. This threatens epithelial integrity and homeostasis of the gut [15]. In addition, CD4^+^Foxp3^+^ Treg cells were found to be upregulated by *Lactobacillus reuteri* in the intestine of mice with experimental necrotizing enterocolitis [10]. To date, the aryl hydrocarbon receptor (AhR) signaling pathway and the serotonin (5-HT) receptors have been proven to play essential roles in the modulation of gut inflammation [16,17]. However, further studies are needed to explore their regulation and mechanisms during gut inflammation.

In gut inflammation, the self-renewal and differentiation mechanisms of the intestinal stem cells (ISCs) are important as they help to replenish damaged cells in the villi [18]. Previous evidence has shown that damage repair of the gut epithelium is an important target for intestinal inflammation, which means that repairing the function of the gut barrier (e.g., immune function and tight junction) could be a treatment for intestinal inflammation [19]. There are many key receptors and signaling molecules that regulate the regeneration of the intestinal epithelium. First, the expression of leucine-rich repeat-containing G-protein-coupled receptor 5 (Lgr5) expression on ISCs regulates the generation of many functional intestinal cells such as enterocytes, enteroendocrine cells, goblet cells, and Paneth cells [20]. In addition, Lgr5 and Ki67 are considered to be markers of epithelial proliferation, which is needed for the repair of intestinal mucosal cells [21]. In addition, the transforming growth factor-β (TGF-β) protein family is involved in gut inflammation and regulates the proliferation and differentiation of cells [22].

The gut microbiota and its metabolites have been shown to play important roles in the development and recovery of gut inflammation and related metabolic disorders [23,24,25]. Notably, the microbial indole metabolites produced from tryptophan are ligands of AhR, which regulates the gut barrier and immune function [26]. It has been found that *Lactobacillus reuteri*, in combination with a diet rich in tryptophan, promotes the maturation and function of immunoregulatory T cells [27]. *Limosilactobacillus mucosae* (LM) was isolated from the intestines of both humans and piglets and has been found to alleviate neuro-psychiatric disorders by suppressing intestinal dysbiosis [28,29,30]. LM possesses a colonization factor gene called the mub gene, which is similar to the genes responsible for the adhesive capacity of *Lactobacillus reuteri* [30]. Additionally, LM has been found to process genes that confer bile salt tolerance and high survival rates in the stomach, indicating its potential use as a probiotic bacterium [31]. Our recent findings showed that tryptophan supplementation in the culture medium increased the production of indole-3-lactic acid and indole-3-acetic acid (IAA) by LM [32]. LM alleviates experimental colitis in mice through the regulation of colonic macrophage function via the TGF-β/5-HT receptor (HTR) pathways [33].

In recent decades, studies on the health-promoting effects of postbiotics (i.e., cell components and metabolites from probiotic bacteria) have shown that they also harbor probiotic effects but have increased stability and safety properties [34]. It was found that heat-killed components of the probiotic bacteria *Lactobacillus rhamnosus* and *Lactobacillus plantarum* and bacteria-derived proteins alleviate gut inflammation by reducing the levels of the pro-inflammatory cytokine IL-18 and increasing the levels of the anti-inflammatory cytokine IL-10 [35]. Heat-killed *L*. *rhamnosus* protects against colitis-induced increases in the permeability of the mucosal barrier [36]. In addition, a study with mice showed that the culture supernatant of *L*. *reuteri* attenuates lipopolysaccharide (LPS)-induced acute liver injury [37]. However, to improve the efficacy and stability of probiotics, it is critical to identify differences in the mode of action of the probiotic effects of different forms and components of certain species of lactobacilli.

This study aims to identify the differences in the mechanisms of various forms and components of *Limosilactobacillus mucosae* in the alleviation of gut inflammation using the dextran sodium sulfate (DSS)-induced colitis mouse model. The results of this study may be used to optimize the functionality of probiotic bacteria to develop new dietary approaches that target gut function in both humans and animals.

## 2. Materials and Methods

### 2.1. Materials

DSS (Cat# 160110) was acquired from MP Biomedicals (Shanghai, China). The primary antibodies for tryptophan hydroxylase 1 (Tph1) (Cat# 12339), phospho-STAT3 (Cat# 9145), and the signal transducer and activator of transcription 3 (STAT3) (Cat# 9139) were purchased from Cell Signaling Technology. Santa Cruz Biotechnology provided the primary antibody for glyceraldehyde 3-phosphate dehydrogenase (GAPDH) (Cat# sc-32233). Proteintech (Wuhan, China) supplied the serotonin receptor 4 (HTR4) primary antibody (Cat# 21165). Unless otherwise specified, all other chemicals were purchased from Merck (Shanghai, China).

### 2.2. Bacterial Strains and Culture Conditions

The *Limosilactobacillus mucosae* (LM) that was studied in this investigation was previously isolated from the small intestines of piglets in our laboratory; this is a dominant *Lactobacillus* in the small intestine of piglets [32]. Stock cultures of LM were reactivated and cultured in Hungate tubes at 37 °C in de Man, Rogosa, and Sharp (MRS) broth overnight before use. The number of viable LM in the tubes was determined via the plate counting of the 18 h culture and the subsequent calculation of colony-forming units (CFU).

For the preparation of different forms and components of LM, the LM was subcultured in MRS broth overnight (18 h) to achieve a bacterial density of 1 × 10^9^ CFU/mL. LM was prepared by centrifugation of the bacterial culture at 2000× *g* for 5 min to remove any supernatant. The number of live LM was adjusted by mixing the fresh bacterial cells with PBS (sterile phosphate-buffered saline, pH = 7.4), and heat-killed LM (HKLM) was obtained by heating the resulting LM PBS solution at 100 °C for 30 min. The cell-free culture supernatant of LM (LMCS) was prepared by filtering the resulting supernatant of LM cultures through a 0.22 μm syringe filter. The above three forms and components of LM were freshly prepared on the day of treatment.

### 2.3. Animals and Experimental Design

A total of seventy-two male C57BL/6 mice (average body weight of 16.0 g) were used in the experiment (Vital River, Beijing, China). Before delivery, the supplier mixed mice from different litters to avoid the litter effect. The mice were kept in standard mouse cages that were maintained in a specific pathogen-free facility with a room temperature of 22 ± 2 °C and a 12-h light/dark cycle. The mice had free access to water and feed (No. 1022 diet, HFK Biotechnology, Beijing, China) [17]. Following seven days of adaptation, the mice were divided randomly into six groups: control (Con), DSS (2.5% *w*/*v* in water), LM+DSS, HKLM+DSS, LMCS+DSS, and the MRS medium + DSS group (MRS+DSS). The DSS solutions were changed every other day. The mice were gavaged every other day for the first three weeks of the experiment with 100 µL of PBS, live LM in PBS (1 × 10^9^ CFU/mL), and heat-killed LM in PBS, LMCS, or MRS, respectively. From Day 15 to Day 21, DSS was used to induce colitis. In accordance with the protocols of our earlier investigations, the mice were weighed and then killed on Day 23 to measure their organs and take samples [17,33]. In the current study, mice that were dead or with disease were excluded from sampling. Eight mice were randomly selected from each group for the analysis of gene expression and protein abundance of specific proteins and cell sorting. For the ethical approval of the experimental procedures using mice, the Institutional Animal Care and Use Committee of China Agricultural University approved this study (approval No.: AW11013202-1-4, 11 October 2023).

### 2.4. Measurement of Disease Activity Index

The disease activity index (DAI) was applied to assess the severity of colitis according to the previous method [33]. In brief, the body weight loss, fecal blood content, and stool consistency were taken to determine the DAI score as previously described by Park and colleagues [38].

### 2.5. Quantitative RT PCR

Tissues of the colon were ground with a mortar in liquid nitrogen. We used TRIzol reagent (Aidlab Biotechnologies, Beijing, China) to extract the total RNA from the colon. HifairII First Strand cDNA Synthesis SuperMix (Yeasen Biotechnologies, Beijing, China) was used to synthesize cDNA in accordance with the manufacturer’s protocol. We used the ABI 7500 real-time PCR detection system (Applied Biosystems, Dublin, CA, USA) to quantify the gene expression. GAPDH was used as the internal reference when calculating the gene expression via the 2^−ΔΔCt^ method. The sequences of the primers used in this study are shown in Supplemental Table S1.

### 2.6. Western Blot Analysis

The proteins in the tissues of the colon were extracted using radioimmunoprecipitation assay lysis buffer (RIPA) supplemented with phosphatase and protease inhibitors (MedChemExpress, Beijing, China). The concentrations of the extracted proteins were quantified using a bicinchoninic acid (BCA) assay. An equal amount of extracted protein was separated using 10% SDS–PAGE and then transblotted to PVDF membranes. The membranes were incubated with primary antibodies against pSTAT3, STAT3, Tph1, and AhR overnight at 4 °C, followed by incubation with secondary antibodies. The chemiluminescence of the bands was visualized on an ImageQuant LAS 4000 (GE Healthcare, Hanover, PA, USA). The quantification of the band density was performed using ImageJ version 1.54h (NIH). The internal reference was GAPDH.

### 2.7. Isolation and Analysis of Immune Cells in the Spleen Using Flow Cytometry

We isolated the immune cells from the spleen of mice using a cell strainer and the Percoll gradient centrifugation method as previously described. For the incubation of different antibodies for cell-sorting of different immune cells, three panels were used in the experiment: panel 1 (CD45, CD3, and CD4), panel 2 (CD45, CD64, CD11b, and Ly6G), and panel 3 (CD45, CD4, CD3, and FOXP3). For the arrangement of the channels for cell sorting of the cells in each tube, the PC-A750, APC, PC5.5, and FITC channels were used for cell sorting in tube 1; the APC-A750, PC5.5, APC, and PE channels were used for cell sorting in tube 2; and the PC5.5, APC, APC-A750, and PE channels were used for cell sorting in tube 3. A FACS Canto Flow Cytometer (Beckman Coulter, Brea, CA, USA) was used for flow cytometry analysis. The percentage (%) of the total CD45^+^ populations represents the abundances of specific groups of immune cells, such as CD11b^+^Ly6G^+^ neutrophils, CD11b^+^CD64^+^ macrophages, CD4^+^Foxp3^+^ regulatory T cells, CD3^+^CD4^+^ T helper cells, and CD3^+^CD8^+^ cytotoxic T cells. The CytExpert version 2.3 (Beckman Coulter) was used for the analysis of the flow cytometry data.

### 2.8. Analysis of 5-HT and Trp Metabolites

The colon tissues and fecal samples were weighed, homogenized, and deproteinized with extraction solution [39]. After the deproteinization of the samples, we diluted the supernatant with extraction solution, and reverse-phase HPLC was used to determine the concentrations of Trp metabolites; this included 5-HT, indole-3-lactic acid (ILA), indole-3-propionic acid (IPA), indole-3-acetic acid (IAA), indole, 5-hydroxyindoleacetic acid (5-OH-IAA), and 3-methylindole [39].

### 2.9. Statistical Analysis

The number of animals required to evaluate the effect of different forms and components of LM on colitis was based on our previous study [33]. The sample size of this study was estimated using G*Power 3.1.9.6 (RRID: SCR_013726) F tests [40,41] with a setting of α = 0.05, power = 0.95, and SD within each group set as 10 (effect size f calculated as 1.15) based on our preliminary study on DSS-induced body weight change (original 100) of mice (*n* = 8). The calculated results showed that a sample size greater than 5 per group was sufficient to find statistically significant differences between groups with an actual power of 0.98. In the current study, we used SPSS 16.0 (IBM Inc., New York, NY, USA) for statistical analysis. Differences among the experimental groups were analyzed using one-way ANOVA followed by Duncan’s multiple comparison procedure. Means ± standard error of means (SEMs) are used to express the data. A *p*-value < 0.05 was used to indicate statistically significant values.

## 3. Results

### 3.1. Effects of Different Forms and Components of LM on Body Weight Change, DAI Score, Colon Length, and Spleen Index

**Figure 1:**
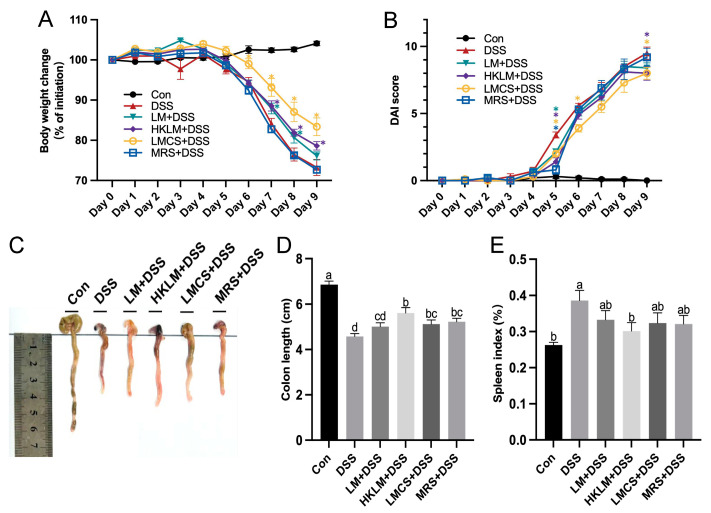
Effects of different forms and components of LM on the body weight change (**A**), disease activity index (**B**), colon length (**C**,**D**), and spleen index (**E**) of mice treated with DSS. Data in the charts are means ± SEMs, *n* = 12. * *p* < 0.05 compared to the DSS group. a–d, means in the charts with different letters differ, *p* < 0.05. Con, control; DAI, disease activity index; DSS, dextran sodium sulfate; HKLM, heat-killed *Limosilactobacillus mucosae*; LM, *Limosilactobacillus mucosae*; LMCS, *Limosilactobacillus mucosae* culture supernatant.

Three forms and components of LM reduced (*p* < 0.05) the DSS-induced reduction in body weight and increase in DAI score in mice 8 days after DSS treatment (Figure 1A,B), whereby LMCS differed significantly (*p* < 0.05) from the LM+DSS and HKLM+DSS groups in alleviating the DSS-induced bodyweight loss at Day 6, Day 8, and Day 9 after DSS treatment and the increase in the DAI score at Day 6 after DSS treatment. Mice in the HKLM+DSS group had longer (*p* < 0.05) colons and lower spleen indexes than those in the other DSS treatment groups (Figure 1C–E).

### 3.2. LM, HKLM, and LMCS Differentially Regulated the Expression of Inflammatory Cytokines and Signaling in the Colon

**Figure 2:**
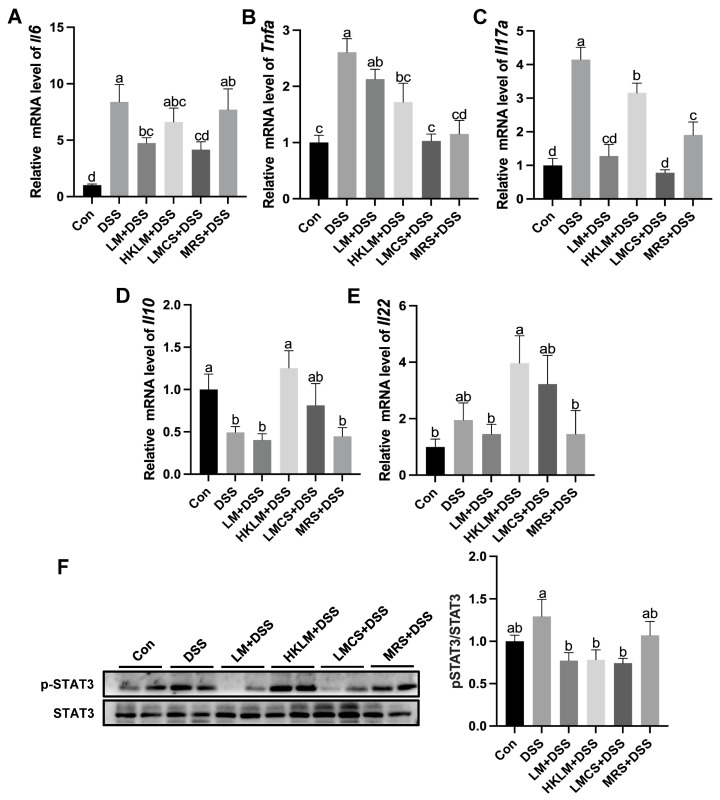
Effects of different forms and components of LM on the expression of inflammatory cytokines *Il6* (**A**), *Tnfa* (**B**), *Il17a* (**C**), *Il10* (**D**), *Il22* (**E**), and ratios of p-STAT3/STAT3 (**F**) in the colon of mice treated with DSS. Data in the charts are means ± SEMs, *n* = 8. a–d, means in the charts with different letters differ, *p* < 0.05.

Compared to the control, mice in the DSS group had higher (*p* < 0.05) mRNA levels of *Il6*, *Tnfa*, and *Il17a* (Figure 2A–C) and lower (*p* < 0.05) mRNA levels of *Il10* (Figure 2D). Compared to the DSS and MRS+DSS groups, mice in the LM+DSS and LMCS+DSS groups had lower (*p* < 0.05) mRNA levels of *Il6* and *Il17a* (Figure 2A,C). HKLM treatment increased (*p* < 0.05) the mRNA levels of *Il10* compared to the DSS and MRS+DSS groups (Figure 2D). Compared to the LM+DSS and LMCS+DSS groups, mice in the HKLM group had higher (*p* < 0.05) mRNA levels of *Il17a* (Figure 2C). Compared to DSS treatment, three forms and components of LM treatment reduced (*p* < 0.05) the ratio of p-STAT3/STAT3 (Figure 2F). Compared to the DSS group, mice in the MRS+DSS group had lower (*p* < 0.05) mRNA levels of *Tnfa* and *Il17a* (Figure 2B,C).

### 3.3. Effects of Different Forms and Components of LM on the Abundances of Immune Cells in the Spleen

**Figure 3:**
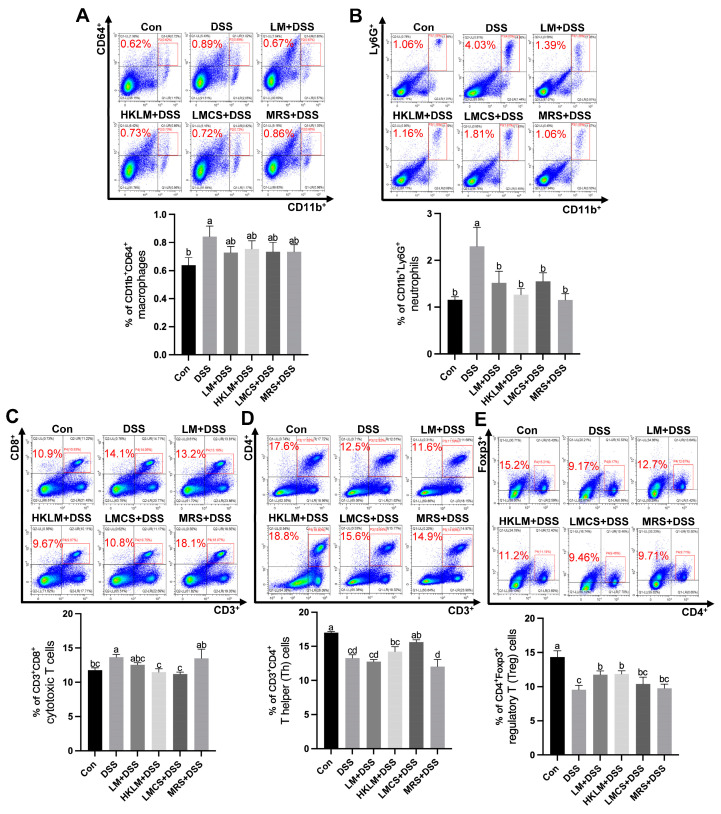
Effects of different forms and components of LM on the abundances of macrophages (**A**), neutrophils (**B**), cytotoxic T cells (**C**), T helper cells (**D**), and regulatory T cells (**E**) in the spleen of mice treated with DSS. Values with red color in the representative flow cytometry diagrams are percentages of target immune cells to CD45^+^ populations. Data in the charts are means ± SEMs, *n* = 8. a–d, means in the charts with different letters differ, *p* < 0.05.

In the spleen, compared to the control, mice in the DSS group had higher (*p* < 0.05) percentages of CD11b^+^Ly6G^+^ neutrophils, CD11b^+^CD64^+^ macrophages, and CD3^+^CD8^+^ cytotoxic T cells but lower (*p* < 0.05) percentages of CD4^+^Foxp3^+^ regulatory T cells and CD3^+^CD4^+^ T helper cells (Figure 3). Compared to the DSS and MRS+DSS groups, mice in the HKLM+DSS and LMCS+DSS groups had lower (*p* < 0.05) percentages of CD3^+^CD8^+^ cytotoxic T cells (Figure 3C) but higher (*p* < 0.05) percentages of CD3^+^CD4^+^ T helper cells (Figure 3D). In addition, compared to the DSS group, the administration of LM or HKLM increased (*p* < 0.05) the percentages of CD4^+^Foxp3^+^ regulatory T cells (Figure 3E). Compared to the DSS group, mice in the MRS+DSS group had lower (*p* < 0.05) abundances of CD11b^+^Ly6G^+^ neutrophils (Figure 3A,B).

### 3.4. Effects of Different Forms and Components of LM on the Expression of Lgr5, Ki67, and Tgfb1 in the Colon

**Figure 4:**
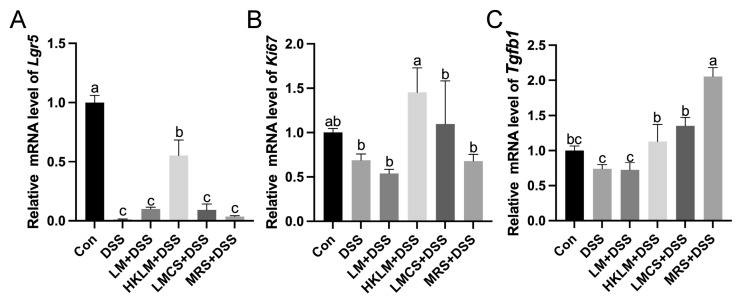
Effects of different forms and components of LM on the expression of cell proliferation- and differentiation-related genes *Lgr5* (**A**), *Ki67* (**B**), and *Tgfb1* (**C**) in the colon of mice treated with DSS. Data in the charts are means ± SEMs, *n* = 8. a–c, means in the charts with different letters differ, *p* < 0.05.

In the colon, compared to the control, DSS treatment reduced (*p* < 0.05) the mRNA levels of *Lgr5* (Figure 4A) and showed a tendency to reduce (0.1 < *p* < 0.05) the mRNA levels of *Ki67* and *Tgfb1* (Figure 4B,C). Compared to the DSS and MRS+DSS groups, mice in the HKLM group had higher (*p* < 0.05) mRNA levels of *Lgr5* and *Ki67* in the colon (Figure 4A,B). Compared to the DSS group, mice in the HKLM+DSS, LMCS+DSS, and MRS+DSS groups had higher (*p* < 0.05) mRNA levels of *Tgfb1* (Figure 4C). In addition, mice in the MRS+DSS group had the highest (*p* < 0.05) mRNA levels of *Tgfb1* compared to the other groups (Figure 4C).

### 3.5. LM, HKLM, and LMCS Differentially Regulated the Expression of HTRs in the Colon

**Figure 5:**
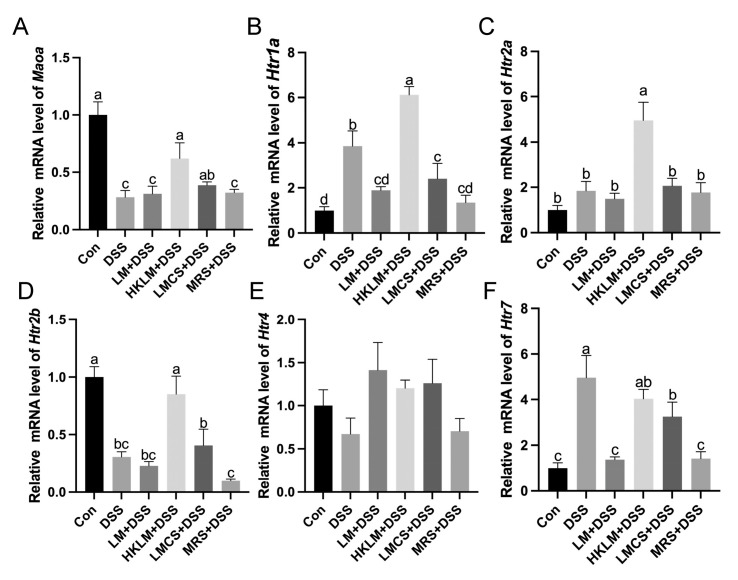
Effects of different forms and components of LM on the expression of 5-HT degradation gene *Maoa* (**A**) and 5-HT receptors *Htr1a* (**B**), *Htr2a* (**C**), *Htr2b* (**D**), *Htr4* (**E**), and *Htr7* (**F**) in the colon of mice treated with DSS. Data in the charts are means ± SEMs, *n* = 8. a–d, means in the charts with different letters differ, *p* < 0.05.

Compared to the control, mice in the DSS group had higher (*p* < 0.05) mRNA levels of *Htr1a* and *Htr7* (Figure 5B,F) but lower (*p* < 0.05) mRNA levels of *Htr2b* in the colon (Figure 5D). Compared to the DSS and MRS+DSS groups, mice in the HKLM group had higher (*p* < 0.05) mRNA levels of *Htr2a* and *Htr2b* in the colon (Figure 5C,D). Three different forms and components of LM showed a tendency to increase (0.1 < *p* < 0.05) the mRNA levels of *Htr4* in the colon compared to the DSS and MRS+DSS groups (Figure 5E). In addition, compared to the DSS group, mice in the MRS+DSS group had lower (*p* < 0.05) mRNA levels of *Htr1a* and *Htr7* in the colon (Figure 5B,F).

### 3.6. LM, HKLM, and LMCS Differentially Regulated Levels of 5-HT and Indoles in the Colon

**Figure 6:**
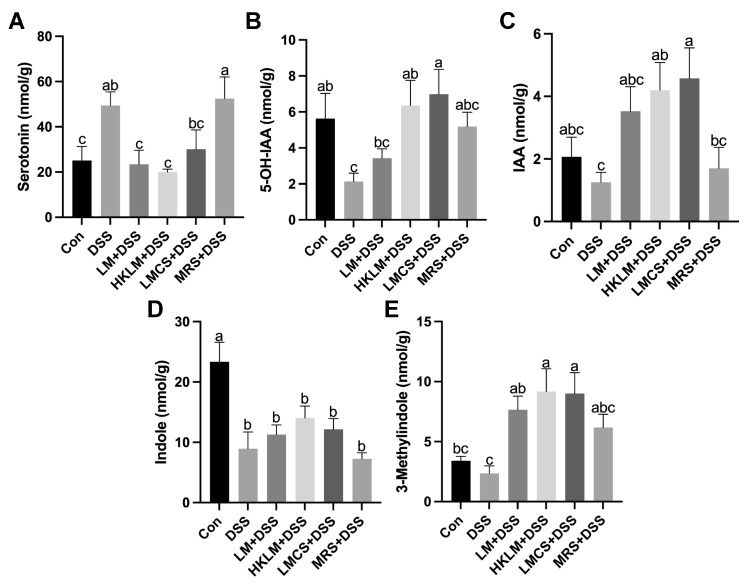
Effects of different forms and components of LM on Trp metabolites serotonin (**A**), 5-OH-IAA (**B**), IAA (**C**), indole (**D**), and 3-methylindole (**E**) in the colon of mice treated with DSS. Data in the charts are means ± SEMs, *n* = 8. a–c, means in the charts with different letters differ, *p* < 0.05.

In the colon, compared to the control, DSS treatment increased (*p* < 0.05) the levels of 5-HT but reduced (*p* < 0.05) the levels of 5-OH-IAA (Figure 6A,B). LM or LMCS treatment increased (*p* < 0.05) DSS-induced reduction of concentrations of IAA and indole in the feces (Supplemental Table S2). Our analysis also showed that LMCS and MRS have high levels of 5-OH-IAA and ILA (Supplemental Table S3). Mice in the DSS group showed a tendency to possess an increased (0.1 < *p* < 0.05) protein abundance of Tph1 (Supplemental Figure S1A) but a downregulated (*p* < 0.05) expression of *Maoa* in the colon compared to the control (Figure 5A). For microbial indole metabolites, compared to the control, DSS treatment reduced (*p* < 0.05) the levels of indole in the colon (Figure 6A,B,D; Supplemental Table S2).

In addition, DSS treatment reduced (*p* < 0.05) the protein abundance of AhR in the colon compared to the control (Supplemental Figure S1B). Compared to the DSS group, mice in the LMCS+DSS and MRS+DSS groups had higher (*p* < 0.05) protein abundances of AhR in the colon (Supplemental Figure S1B).

## 4. Discussion

*Lactobacillus* species are a key gut microbial community that plays a vital role in the modulation of the composition and metabolism of gut microbiota in disease and health. However, the efficacy and mechanisms of the probiotic effects of *Lactobacillus* species are determined by many factors, such as different forms and components (live, heat-killed, or cell-free culture supernatant), different cell components, and different bacterial cells-derived metabolites [34]. A previous meta-analysis has shown that heat-killed probiotics, statistically, have the same ability to relieve colitis as living ones [42]. Considering the limitations of the use of live probiotics, here we investigated the variations in the efficacy and mechanisms of different forms and components of LM (live, heat-killed, and cell-free supernatant) in the alleviation of DSS-induced colon inflammation in mice. The current study discovered the following: (1) LMCS was effective in mitigating the body weight loss of mice after treatment with DSS; (2) all three forms and components of LM treatments increased the indole-3-acetic acid levels and reduced the 5-HT levels in the colon; (3) LM or HKLM increased the abundance of splenic CD4^+^Foxp3^+^ Treg cells; and (4) LM and LMCS reduced the expression of the pro-inflammatory cytokines *Il6* and *Il17a*, but HKLM upregulated anti-inflammatory cytokine *Il10*, stem cell proliferation and differentiation markers (*Lgr5* and *Ki67*), the 5-HT degradation enzyme *Maoa*, and 5-HT receptors (*Htr1a*, *Htr2a*, and *Htr2b*) in the colon of mice with DSS-induced inflammation. The above findings help to further explain the probiotic effects and mechanisms of different forms and components of gut lactobacilli, such as LM.

The findings of our current study suggest that the differential regulation of the homeostasis of the pro-inflammatory and anti-inflammatory cytokines with an increased abundance of Treg cells is an important probiotic mechanism of LM. Gut inflammation shows many pathophysiological features, such as oxidative damage, immune activation, cytokine dysregulation, and apoptosis [43]. Naive CD4^+^ lymphocytes undergo differentiation into different regulatory or effector cells after inflammation [44]. As one type of differentiated T cells, Th17 cells can produce IL-17A and IL-22 and play an important role in the immune response in gut inflammation [45]. In addition, Treg cells are one of the major cell types that produce the anti-inflammatory cytokine IL-10 [22]. Meanwhile, TNF-α and IL-6, which are produced by macrophages and T cells, enhance the activation of macrophages and the presentation of antigens [46]. Consistent with the above study, we found that high levels of macrophages and neutral cells were observed in the groups with a high expression of IL-6 and TNF-α. Similarly, in the groups with a low expression of TNF-α and IL-6, the expression levels of the aforementioned immune cells were also low. This result suggested that the reduced abundance of macrophages and cytotoxic T cells that was caused by LMCS was associated with the reduced expression of *Il6* and *Il17a*. This accords with the fact that the changes in the characteristics of specific subtypes of immune cells might result in the increased production of inflammatory cytokines such as IL-6 and IL-17A [47]. This also blocks the activation of STAT3, which can promote tumorigenesis in this model [48]. Strikingly, treatment with MRS medium before and during DSS treatment also reduced the numbers of macrophages and neutrophils in the current study after DSS treatment. A possible explanation for the ability of MRS medium to reduce the number of macrophages in the colon during inflammation is that MRS medium is rich in amino acids and contains functional oligosaccharides in the yeast extract of the formula, which modulates gut function. As such, we included the MRS medium in this study in order to determine that the effects of the cell-free supernatant were not due to components in the MRS medium. Further research is warranted to understand the detailed mechanisms of the various components of probiotic bacteria in the regulation of immune cell function, either through the inhibition of pro-inflammatory cytokines or the activation of anti-inflammatory cytokines in the intestine.

Our findings also suggest the possible importance of the interactions between 5-HT signaling and epithelial cell renewal in the inflamed intestine. However, it is generally accepted that the limited presence of 5-HT in the digestive tract exerts various physiological effects by binding to different receptors [49]. Gut HTRs play both anti-inflammatory and pro-inflammatory roles in gut inflammation [50]. Enteritis that is chemically induced by DSS or acetic acid has been associated with the activation of pro-inflammatory HTRs such as HTR2A and HTR7 [17,51]. Nevertheless, the activation of HTRs that reduce gut inflammation, such as HTR4 and HTR2B, via dietary interventions has been found to alleviate gut inflammation [17]. It is generally accepted that the self-renewal and differentiation of ISCs are important during wound healing in the gut epithelium, as the replenishment of damaged cells in the villi is necessary for the prevention of secondary tissue injury [18]. In the current study, pretreatment with HKLM upregulated the expression of Lgr5 and Ki67. Interestingly, mice treated with HKLM, LMCS, or MRS have higher levels of colonic TGF-β1, which may promote the proliferation of epithelial cells to aid in tissue repair. Our previous findings with mice showed that tryptophan (Trp) or LM differentially regulated the family of TGF-β protein expression and *Htr4* expression, suggesting that HTRs possibly play an important role in the regulation of gut epithelial cell renewal [17,33]. Further studies are required to uncover these detailed mechanisms.

In addition, the tryptophan metabolite repertoire in the colon may be an important factor in the regulation of gut inflammation [6]. The intestinal microbiota influences the physiological functions of the gut via metabolites that are produced by the gut microbiota and are derived from the transformation of molecules of environmental origin [52]. A few commensal species are able to directly transform Trp into bioactive molecules, including indole and its derivatives. Several studies have also found that the inoculation of mice with three Trp-metabolizing *Lactobacillus* strains attenuates intestinal inflammation. In addition, another pathway being extensively studied with regard to the Trp metabolism involved is the pathway by which enterochromaffin cells produce 5-HT via *Tph1* [53], which is the key topic discussed in this paper. It has been shown that IBD is associated with the increased expression of *Tph1* and higher levels of 5-HT [54]. Consistent with the above results, in the current study, we found that LM reversed the DSS-induced upregulation of Tph1 and increased the levels of 5-HT in the colon. This is achieved via the upregulation of MAOA, which is the main enzyme that converts 5-HT to 5-OH-IAA [55]. Furthermore, after the inoculation of mice with *Lactobacillus* strains that possess the ability to metabolize tryptophan, a noticeable reduction in gut inflammation is observed [32,33,56]. However, these findings require further confirmation.

Our findings also suggest the importance of identifying the properties and mechanisms of specific forms/components of probiotic bacteria in the development of new parabiotics, postbiotics, and their combination. A study with *Lactobacillus paracasei* showed that viable cells and heat-killed cells, but not cell-free supernatant, suppress LPS-induced TNF-α secretion in Caco-2 cells. However, the authors only tested the efficacy of live *L*. *paracasei* in the alleviation of DSS-induced colitis in rats [57]. A study using rats with metabolic syndrome showed that heat-killed *L*. *plantarum* L-137 attenuated the inflammation and fibrosis of the left ventricle, which was partially due to its upregulation of the abundance of splenic Treg cells [58]. In addition, a study on the skin wound-healing effects of *L*. *plantarum* KB131 showed that heat-killed *L*. *plantarum* KB131 accelerated wound closure and increased the synthesis of C-C motif chemokine ligand 5 (CCL5) and the abundance of M2 macrophages in the wound tissues [59]. A study comparing the efficacy of the cell-free supernatant from different *Lactobacillus* species (i.e., *Lactobacillus acidophilus*, *L. plantarum*, *Lactobacillus rhamnosus*, and *Lactobacillus casei*) showed that the cell-free supernatant of *L*. *acidophilus* increased the Cl^−^/HCO_3_^−^ exchange activity in Caco-2 cells [60]. Both the live and cell-free supernatant of *L*. *acidophilus* attenuated the interferon-γ-induced decrease in the Cl^−^/HCO_3_^−^ exchange activity in Caco-2 cells [60]. Additionally, the comparison of different forms of *Lactobacillus fermentum* in the alleviation of *Yersinia enterocolitica*-induced inflammation showed that viable cells and the supernatant, but not heat-killed cells, inhibited IL-8 secretion and inhibited the activation of the NF-κB signaling pathway in HeLa cells [61]. However, the treatment using the supernatant of *L*. *fermentum* with phospholipase deteriorated the above effects, suggesting that the phospholipid secreted in the medium mediated the anti-inflammatory effects of *L*. *fermentum* [61]. In addition, a recent study revealed that the stress protein chaperonin group I protein (GroEL or Cpn60), secreted by *L*. *reuteri* but not from *Escherichia coli*, inhibited the polarization of the pro-inflammatory M1 macrophage and cytokine production in human macrophages [62]. The above findings suggested that *Lactobacillus*-secreted proteins and metabolites are important for their immune-modulating effects. Interestingly, our recent studies showed that Trp increased the production of the immune-modulatory metabolites 3-indoleacetic acid and indole-3-lactic acid via LM in the culture media [32]. This may partially explain the probiotic effects of LM in the gut immune response [33]. However, the mechanisms implicated in the regulation of the 5-HT-mediated gut immune function require further investigation [6].

The mechanisms of different forms and components of probiotic bacteria implicated in the regulation of the gut immune response are complex and differ due to probiotic bacteria harboring the ability to regulate the metabolism and physiology in both host cells and the gut microbiota. In the current study, live LM and LMCS were prone to downregulating the expression of pro-inflammatory cytokines and HTRs. However, HKLM upregulated anti-inflammatory cytokines and HTRs; they did not alter the expression of the pro-inflammatory cytokines and HTRs upregulated by DSS treatment. This indicates that the live bacteria in the intestine may exert their immuno-modulatory effects through their secretions and that HKLM exerts anti-inflammatory effects via different routes. It is possible that after high-temperature treatment, intracellular active substances are released or that high temperatures alter the structure of the bioactive components that regulate gut immune homeostasis. These hypotheses still need further investigation and validation.

This study also has some limitations. First, the specific components of HKLM and LMCS that alleviate colon inflammation in mice have not been clearly identified. Second, whether LM regulates the composition of the gut microbiota and the production of microbial metabolites other than indoles in both males and females was not considered. Third, this study could not distinguish whether the differences in the levels of indole metabolites in the colon were of LM origin or produced by other LM-regulated gut microbial communities. It has been shown that the bile metabolites and cell components of LM harbor immunomodulatory effects. The purified exopolysaccharide and secretome derived from the dead cells and the cell-free supernatant of LM strains DPC 6426 upregulate the expression of *Il6* and *Il10* in murine macrophages, while the entire secretome was necessary to increase the expressions of markers for anti-inflammatory M2 macrophage polarization [63]. Further studies are warranted to discover the detailed mechanisms implicated in the cell components of LM and their stimulation of the production of anti-inflammatory microbial-derived indole metabolites in the intestines of different genders during inflammation [64].

## 5. Conclusions

In conclusion, three forms and components of LM increased the levels of indole-3-acetic acid in the colon and the abundance of Treg cells in the spleen but reduced the levels of serotonin in the colon. Viable LM or its cell-free supernatant reduced the expression of the pro-inflammatory cytokines *Il6* and *Il17a*. Heat-killed LM increased the expression of the anti-inflammatory cytokine *Il10*, the gut stem cell proliferation and differentiation genes *Lgr5* and *Ki67*, and the serotonin receptors *Htr1a*, *Htr2a*, and *Htr2b* in the colon. These findings suggested that tryptophan metabolism and serotonin signaling, coupled with their regulation of stem cell differentiation and proliferation in the intestine, are important pathways for the immune-modulating effects of probiotic bacteria (e.g., LM) in the prevention and alleviation of inflammatory gut disorders.

## Data Availability

All data presented in the study were included in the article.

## Supplementary Materials

The following supporting information can be downloaded at: https://www.mdpi.com/article/10.3390/nu16040468/s1, Figure S1: Effects of different forms and components of LM treatment on the protein abundance of Tph1 (A) and AhR (B) in the colon of mice treated with DSS; Table S1: Sequences of primers used in this study; Table S2: Effects of different forms and components of LM administration and DSS treatment on concentrations of tryptophan metabolites in the feces of mice; Table S3: Concentrations of tryptophan metabolites in HKLM supernatant, LMCS, and MRS medium.

## References

[1] Guo C., Kong L., Xiao L., Liu K., Cui H., Xin Q., Gu X., Jiang C., Wu J. (2023). The impact of the gut microbiome on tumor immunotherapy: From mechanism to application strategies. Cell Biosci..

[2] Liang H., Dai Z., Liu N., Ji Y., Chen J., Zhang Y., Yang Y., Li J., Wu Z., Wu G. (2018). Dietary L-tryptophan modulates the structural and functional composition of the intestinal microbiome in weaned piglets. Front. Microbiol..

[3] Ayyanna R., Ankaiah D., Arul V. (2018). Anti-inflammatory and antioxidant properties of probiotic bacterium *Lactobacillus mucosae* AN1 and *Lactobacillus fermentum* SNR1 in Wistar Albino rats. Front. Microbiol..

[4] Xue D., Cheng Y., Pang T., Kuai Y., An Y., Wu K., Li Y., Lai M., Wang B., Wang S. (2023). Sodium butyrate alleviates deoxynivalenol-induced porcine intestinal barrier disruption by promoting mitochondrial homeostasis via PCK2 signaling. J. Hazard. Mater..

[5] Aljada B., Zohni A., El-Matary W. (2021). The gluten-free diet for celiac disease and beyond. Nutrients.

[6] Jiang L., Han D., Hao Y., Song Z., Sun Z., Dai Z. (2023). Linking serotonin homeostasis to gut function: Nutrition, gut microbiota and beyond. Crit. Rev. Food Sci. Nutr..

[7] Hagen M., Pangrazzi L., Rocamora-Reverte L., Weinberger B. (2023). Legend or truth: Mature CD4^+^CD8^+^ double-positive T cells in the periphery in health and disease. Biomedicines.

[8] Shui J.W., Larange A., Kim G., Vela J.L., Zahner S., Cheroutre H., Kronenberg M. (2012). HVEM signalling at mucosal barriers provides host defence against pathogenic bacteria. Nature.

[9] Nancey S., Holvöet S., Graber I., Joubert G., Philippe D., Martin S., Nicolas J.F., Desreumaux P., Flourié B., Kaiserlian D. (2006). CD8^+^ cytotoxic T cells induce relapsing colitis in normal mice. Gastroenterology.

[10] Liu Y., Tran D.Q., Fatheree N.Y., Marc Rhoads J. (2014). *Lactobacillus reuteri* DSM 17938 differentially modulates effector memory T cells and Foxp3+ regulatory T cells in a mouse model of necrotizing enterocolitis. Am. J. Physiol. Gastrointest. Liver Physiol..

[11] Fantini M.C., Monteleone G. (2017). Update on the therapeutic efficacy of Tregs in IBD: Thumbs up or thumbs down?. Inflamm. Bowel Dis..

[12] Gong Y., Liu L., He X., Zhao H., Yang J., Li L., Lu A., Lin Y., Jiang M. (2015). The Th17/Treg immune balance in ulcerative colitis patients with two different Chinese syndromes: Dampness-heat in large intestine and spleen and kidney yang deficiency syndrome. Evid. Based Complement. Alternat Med..

[13] Nielsen O.H., Kirman I., Rüdiger N., Hendel J., Vainer B. (2003). Upregulation of interleukin-12 and -17 in active inflammatory bowel disease. Scand. J. Gastroenterol..

[14] Gong Y., Lin Y., Zhao N., He X., Lu A., Wei W., Jiang M. (2016). The Th17/Treg immune imbalance in ulcerative colitis disease in a Chinese Han population. Mediat. Inflamm..

[15] Das I., Png C.W., Oancea I., Hasnain S.Z., Lourie R., Proctor M., Eri R.D., Sheng Y., Crane D.I., Florin T.H. (2013). Glucocorticoids alleviate intestinal ER stress by enhancing protein folding and degradation of misfolded proteins. J. Exp. Med..

[16] Panda S.K., Peng V., Sudan R., Ulezko Antonova A., Di Luccia B., Ohara T.E., Fachi J.L., Grajales-Reyes G.E., Jaeger N., Trsan T. (2023). Repression of the aryl-hydrocarbon receptor prevents oxidative stress and ferroptosis of intestinal intraepithelial lymphocytes. Immunity.

[17] Wang B., Sun S., Liu M., Chen H., Liu N., Wu Z., Wu G., Dai Z. (2020). Dietary L-tryptophan regulates colonic serotonin homeostasis in mice with dextran sodium sulfate-induced colitis. J. Nutr..

[18] Won J.H., Choi J.S., Jun J.I. (2022). CCN1 interacts with integrins to regulate intestinal stem cell proliferation and differentiation. Nat. Commun..

[19] Boal Carvalho P., Cotter J. (2017). Mucosal healing in ulcerative colitis: A comprehensive review. Drugs.

[20] Barker N., van Es J.H., Kuipers J., Kujala P., van den Born M., Cozijnsen M., Haegebarth A., Korving J., Begthel H., Peters P.J. (2007). Identification of stem cells in small intestine and colon by marker gene Lgr5. Nature.

[21] Song S., Bai M., Ling Z., Lin Y., Wang S., Chen Y. (2021). Intermittent administration of a fasting-mimicking diet reduces intestinal inflammation and promotes repair to ameliorate inflammatory bowel disease in mice. J. Nutr. Biochem..

[22] Ihara S., Hirata Y., Koike K. (2017). TGF-β in inflammatory bowel disease: A key regulator of immune cells, epithelium, and the intestinal microbiota. J. Gastroenterol..

[23] Blacher E., Levy M., Tatirovsky E., Elinav E. (2017). Microbiome-modulated metabolites at the interface of host immunity. J. Immunol..

[24] Wang J., Li Y., Cao C., Yang R., He M., Yan J., Huang P., Tan B., Fan Z. (2023). The periparturient gut microbiota’s modifications in shaziling sows concerning bile acids. Metabolites.

[25] Lavelle A., Sokol H. (2020). Gut microbiota-derived metabolites as key actors in inflammatory bowel disease. Nat. Rev. Gastroenterol. Hepatol..

[26] Scott S.A., Fu J., Chang P.V. (2020). Microbial tryptophan metabolites regulate gut barrier function via the aryl hydrocarbon receptor. Proc. Natl. Acad. Sci. USA.

[27] Cervantes-Barragan L., Chai J.N., Tianero M.D., Di Luccia B., Ahern P.P., Merriman J., Cortez V.S., Caparon M.G., Donia M.S., Gilfillan S. (2017). *Lactobacillus reuteri* induces gut intraepithelial CD4^+^CD8αα^+^ T cells. Science.

[28] Kim J.K., Lee K.E., Lee S.A., Jang H.M., Kim D.H. (2020). Interplay between human gut bacteria *Escherichia coli* and *Lactobacillus mucosae* in the occurrence of neuropsychiatric disorders in mice. Front. Immunol..

[29] Han S.K., Kim D.H. (2019). *Lactobacillus mucosae* and *Bifidobacterium longum* synergistically alleviate immobilization stress-induced anxiety/depression in mice by suppressing gut dysbiosis. J. Microbiol. Biotechnol..

[30] Roos S., Karner F., Axelsson L., Jonsson H. (2000). *Lactobacillus mucosae* sp. nov., a new species with in vitro mucus-binding activity isolated from pig intestine. Int. J. Syst. Evol. Microbiol..

[31] de Moraes G.M.D., de Abreu L.R., do Egito A.S., Salles H.O., da Silva L.M.F., Nero L.A., Todorov S.D., Dos Santos K.M.O. (2017). Functional properties of *Lactobacillus mucosae* strains isolated from brazilian goat milk. Probiotics Antimicrob. Proteins.

[32] Wu S., Liu M., Chen H., Song Q., Wu Z., Dai Z. (2022). Tryptophan regulates bile and nitrogen metabolism in two pig gut lactobacilli species in vitro based on metabolomics study. Amino Acids.

[33] Hao Y., Jiang L., Han D., Si D., Sun Z., Wu Z., Dai Z. (2023). *Limosilactobacillus mucosae* and *lactobacillus amylovorus* protect against experimental colitis via upregulation of colonic 5-hydroxytryptamine receptor 4 and transforming growth factor-β2. J. Nutr..

[34] Salminen S., Collado M.C., Endo A., Hill C., Lebeer S., Quigley E.M.M., Sanders M.E., Shamir R., Swann J.R., Szajewska H. (2021). The International Scientific Association of Probiotics and Prebiotics (ISAPP) consensus statement on the definition and scope of postbiotics. Nat. Rev. Gastroenterol. Hepatol..

[35] Magryś A., Pawlik M. (2023). Postbiotic fractions of probiotics *Lactobacillus plantarum* 299v and *Lactobacillus rhamnosus* GG show immune-modulating effects. Cells.

[36] Miyauchi E., Morita H., Tanabe S. (2009). *Lactobacillus rhamnosus* alleviates intestinal barrier dysfunction in part by increasing expression of zonula occludens-1 and myosin light-chain kinase in vivo. J. Dairy. Sci..

[37] Cui Y., Qi S., Zhang W., Mao J., Tang R., Wang C., Liu J., Luo X.M., Wang H. (2019). *Lactobacillus reuteri* ZJ617 culture supernatant attenuates acute liver injury induced in mice by lipopolysaccharide. J. Nutr..

[38] Park Y.H., Kim N., Shim Y.K., Choi Y.J., Nam R.H., Choi Y.J., Ham M.H., Suh J.H., Lee S.M., Lee C.M. (2015). Adequate dextran sodium sulfate-induced colitis model in mice and effective outcome measurement method. J. Cancer Prev..

[39] Dai Z., Sun S., Chen H., Liu M., Zhang L., Wu Z., Li J., Wu G. (2019). Analysis of tryptophan and its metabolites by high-performance liquid chromatography. Methods Mol. Biol..

[40] Faul F., Erdfelder E., Lang A.G., Buchner A. (2007). G*Power 3: A flexible statistical power analysis program for the social, behavioral, and biomedical sciences. Behav. Res. Methods.

[41] Zhang X., Hartmann P. (2023). How to calculate sample size in animal and human studies. Front. Med..

[42] Poaty Ditengou J.I.C., Ahn S.I., Chae B., Choi N.J. (2023). Are heat-killed probiotics more effective than live ones on colon length shortness, disease activity index, and the histological score of an inflammatory bowel disease-induced murine model? A meta-analysis. J. Appl. Microbiol..

[43] Liu T.C., Stappenbeck T.S. (2016). Genetics and pathogenesis of inflammatory bowel disease. Annu. Rev. Pathol..

[44] Yamane H., Paul W.E. (2013). Early signaling events that underlie fate decisions of naive CD4^+^ T cells toward distinct T-helper cell subsets. Immunol. Rev..

[45] Korn T., Bettelli E., Oukka M., Kuchroo V.K. (2009). IL-17 and Th17 cells. Annu. Rev. Immunol..

[46] Wang T., He C. (2020). TNF-α and IL-6: The link between immune and bone system. Curr. Drug Targets.

[47] Amerikanou C., Papada E., Gioxari A., Smyrnioudis I., Kleftaki S.A., Valsamidou E., Bruns V., Banerjee R., Trivella M.G., Milic N. (2021). Mastiha has efficacy in immune-mediated inflammatory diseases through a microRNA-155 Th17 dependent action. Pharmacol. Res..

[48] Hu B., Elinav E., Huber S., Strowig T., Hao L., Hafemann A., Jin C., Wunderlich C., Wunderlich T., Eisenbarth S.C. (2013). Microbiota-induced activation of epithelial IL-6 signaling links inflammasome-driven inflammation with transmissible cancer. Proc. Natl. Acad. Sci. USA.

[49] Hannon J., Hoyer D. (2008). Molecular biology of 5-HT receptors. Behav. Brain Res..

[50] Spohn S.N., Mawe G.M. (2017). Non-conventional features of peripheral serotonin signaling—The gut and beyond. Nat. Rev. Gastroenterol. Hepatol..

[51] Wang B., Cui L., Song Q., Liu M., Kou J., Sun S., Chen H., Shi Y., Wu Z., Dai Z. (2023). Excessive dietary L-tryptophan regulated amino acids metabolism and serotonin signaling in the colon of weaning piglets with acetate-induced gut inflammation. Amino Acids.

[52] Zhang L.S., Davies S.S. (2016). Microbial metabolism of dietary components to bioactive metabolites: Opportunities for new therapeutic interventions. Genome Med..

[53] Yano J.M., Yu K., Donaldson G.P., Shastri G.G., Ann P., Ma L., Nagler C.R., Ismagilov R.F., Mazmanian S.K., Hsiao E.Y. (2015). Indigenous bacteria from the gut microbiota regulate host serotonin biosynthesis. Cell.

[54] Manocha M., Khan W.I. (2012). Serotonin and GI disorders: An update on clinical and experimental studies. Clin. Transl. Gastroenterol..

[55] Gao J., Xiong T., Grabauskas G., Owyang C. (2022). Mucosal serotonin reuptake transporter expression in irritable bowel syndrome is modulated by gut microbiota via mast cell-prostaglandin E2. Gastroenterology.

[56] Lamas B., Richard M.L., Leducq V., Pham H.P., Michel M.L., Da Costa G., Bridonneau C., Jegou S., Hoffmann T.W., Natividad J.M. (2016). CARD9 impacts colitis by altering gut microbiota metabolism of tryptophan into aryl hydrocarbon receptor ligands. Nat. Med..

[57] Ladda B., Jantararussamee C., Pradidarcheep W., Kasorn A., Matsathit U., Taweechotipatr M. (2023). Anti-inflammatory and gut microbiota modulating effects of probiotic *Lactobacillus paracasei* MSMC39-1 on dextran sulfate sodium-induced colitis in rats. Nutrients.

[58] Uchinaka A., Azuma N., Mizumoto H., Nakano S., Minamiya M., Yoneda M., Aoyama K., Komatsu Y., Yamada Y., Murohara T. (2018). Anti-inflammatory effects of heat-killed *Lactobacillus plantarum* L-137 on cardiac and adipose tissue in rats with metabolic syndrome. Sci. Rep..

[59] Ishi S., Kanno E., Tanno H., Kurosaka S., Shoji M., Imai T., Yamaguchi K., Kotsugai K., Niiyama M., Kurachi H. (2023). Cutaneous wound healing promoted by topical administration of heat-killed *Lactobacillus plantarum* KB131 and possible contribution of CARD9-mediated signaling. Sci. Rep..

[60] Singh V., Kumar A., Raheja G., Anbazhagan A.N., Priyamvada S., Saksena S., Jhandier M.N., Gill R.K., Alrefai W.A., Borthakur A. (2014). *Lactobacillus acidophilus* attenuates downregulation of DRA function and expression in inflammatory models. Am. J. Physiol. Gastrointest. Liver Physiol..

[61] Frick J.S., Schenk K., Quitadamo M., Kahl F., Koberle M., Bohn E., Aepfelbacher M., Autenrieth I.B. (2007). *Lactobacillus fermentum* attenuates the proinflammatory effect of *Yersinia enterocolitica* on human epithelial cells. Inflamm. Bowel Dis..

[62] Dias A.M.M., Douhard R., Hermetet F., Regimbeau M., Lopez T.E., Gonzalez D., Masson S., Marcion G., Chaumonnot K., Uyanik B. (2021). *Lactobacillus* stress protein GroEL prevents colonic inflammation. J. Gastroenterol..

[63] Ryan P.M., Stolte E.H., London L.E.E., Wells J.M., Long S.L., Joyce S.A., Gahan C.G.M., Fitzgerald G.F., Ross R.P., Caplice N.M. (2019). *Lactobacillus mucosae* DPC 6426 as a bile-modifying and immunomodulatory microbe. BMC Microbiol..

[64] Lee J.Y., Kim N., Nam R.H., Sohn S.H., Lee S.M., Choi D., Yoon H., Kim Y.S., Lee H.S., Lee D.H. (2017). Probiotics reduce repeated water avoidance stress-induced colonic microinflammation in Wistar rats in a sex-specific manner. PLoS ONE.

