# Supplementary material for: Living, Heat-Killed Limosilactobacillus mucosae and Its Cell-Free Supernatant Differentially Regulate Colonic Serotonin Receptors and Immune Response in Experimental Colitis
Source: Nutrients. 2024 Feb 6;16(4):468. doi: 10.3390/nu16040468 (PMC10893098; doi:10.3390/nu16040468)
Supplement: Supplementary file 1 [file nutrients-16-00468-s001.zip › nutrients-2854224-supplementary.pdf]

Supplemental Materials

Supplemental Table S1. Sequences of primers used in this study.

Genes	Primer Sequence (5'→3')	Product size, bp	Accession Number
Il6	F: CTGATGCTGGTGACAACCAC<br>R: TCCACGATTTCCCAGAGAAC	143	NM_001314054.1
Il10	F: CCAGGGAGATCCTTTGATGA<br>R: AACTGGCCACAGTTTTCAGG	96	NM_010548.2
Il17a	F: ACGTTTCTCAGCAAACTTAC<br>R: CCCCTTTACACCTTCTTTTC	127	NM_010552.3
Il22	F: CCGAGGAGTCAGTGCTAAGG<br>R: CATGTAGGGCTGGAACCTGT	106	XM_006513865.4
Tnfa	F: CCACCACGCTCTTCTGTCTA<br>R: GGTCTGGGCCATAGAACTGA	101	NM_001278601.1
Lgr5	F: CAATGCCTCTCCACACTTCG<br>R: GAAACATCGAACACCTGCGT	159	NM_010195.2
Ki67	F: GCATCGAGTGTGAGCAAGTT<br>R: TGTCCTGCACCTGTTGATCT	160	XM_006507413.5
Tgfb1	F: TCGACATGGAGCTGGTGAAA<br>R: CTGGCGAGCCTTAGTTTGGA	76	XM_036152883.1
Maoa	F: GGAGAAGCCCAGTATCACAGG<br>R: GAACCAAGACATTAATTTTGTATTCTGAC	113	NM_173740.3
Htr1a	F: CCGTGAGAGGAAGACAGTGAAGAC<br>R: GGTTGAGCAGGGAGTTGGAGTAG	176	NM_008308.5
Htr2a	F: GAACCAACCTCTCCTGCGAA<br>R: GGACACTGCCATGATGACCA	146	NM_172812.3
Htr2b	F: GAGGCTCCGAAGTTCAACCA<br>R: CGGCCCGTTCTGCTATATGT	118	XM_006529146.4
Htr4	F: TAATGTTGGGAGGCTGCTGG<br>R: GGGATGTAGAAGGCCACCAC	193	XM_006525683.5
Htr7	F: AAGTTCTCAGGCTTCCCACG<br>R: TTCGCACACTCTTCCACCTC	92	XM_030250756.2
Gapdh	F: ATGGGAAGCTTGTCATCAACG<br>R: AAGACACCAGTAGACTCCACG	115	NM_001411844.1

Supplemental Table S2. Effects of different forms and components of LM administration and DSS treatment on concentrations of tryptophan metabolites in the feces of mice[1].

Trp metabolites (nmol/g)	Con	DSS	LM +DSS	HKLM +DSS	LMCS +DSS	MRS +DSS	Pooled SEM	*P* value
5-OH-IAA	4.60[a]	2.32[b]	4.37[ab]	6.07[a]	5.59[a]	5.21[a]	0.34	< 0.05
ILA	5.47[a]	1.74[c]	2.44[bc]	3.03[bc]	3.85[b]	2.55[bc]	0.25	< 0.05
IAA	1.89[ab]	0.94[c]	1.94[ab]	1.30[bc]	2.11[ab]	1.38[bc]	0.11	< 0.05
IPA	0.95[a]	0.10[c]	0.21[bc]	0.28[bc]	0.48[b]	0.48[b]	0.06	< 0.05
Indole	24.0[a]	8.41[c]	12.3[b]	11.3[b]	12.4[b]	10.6[b]	1.26	< 0.05
3-Methylindole	0.04[b]	0.08[b]	0.06[b]	0.08[b]	0.07[b]	0.20[a]	0.01	< 0.05

[1]Values are means with pooled SEM. a-c, values within a row without a common letter differ, $P < 0.05$.

Con, control; DSS, dextran sodium sulfate; HKLM, heat-killed *Limosilactobacillus mucosae*; IAA, indole-3-acetic acid; ILA, indole-3-lactic acid; IPA, indole-3-propionic acid; LM, *Limosilactobacillus mucosae*; LMCS, culture supernatant of *Limosilactobacillus mucosae*; MRS, de Man, Rogosa, and Sharp medium; 5-OH-IAA, 5-hydroxy-indoleacetic acid; Trp, tryptophan.

Supplemental Table S3. Concentrations of tryptophan metabolites in HKLM supernatant, LMCS, and MRS medium[1].

Trp metabolites (nmol/mL)	HKLM supernatant	LMCS	MRS	Pooled SEM	*P* value
5-OH-IAA	0.15^{b}	10.5^{a}	13.4^{a}	1.72	< 0.05
ILA	0.10^{c}	0.48^{b}	1.02^{a}	0.12	< 0.05
Indole	0.07^{b}	0.18^{a}	0.24^{a}	0.03	< 0.05

[1]Values are means with pooled SEM. a-c, values within a row without a common letter differ, *P* < 0.05.

HKLM, heat-killed *Limosilactobacillus mucosae*; ILA, indole-3-lactic acid; LM, *Limosilactobacillus mucosae*; LMCS, culture supernatant of *Limosilactobacillus mucosae*; MRS, de Man, Rogosa, and Sharp medium; 5-OH-IAA, 5-hydroxy-indoleacetic acid; Trp, tryptophan.

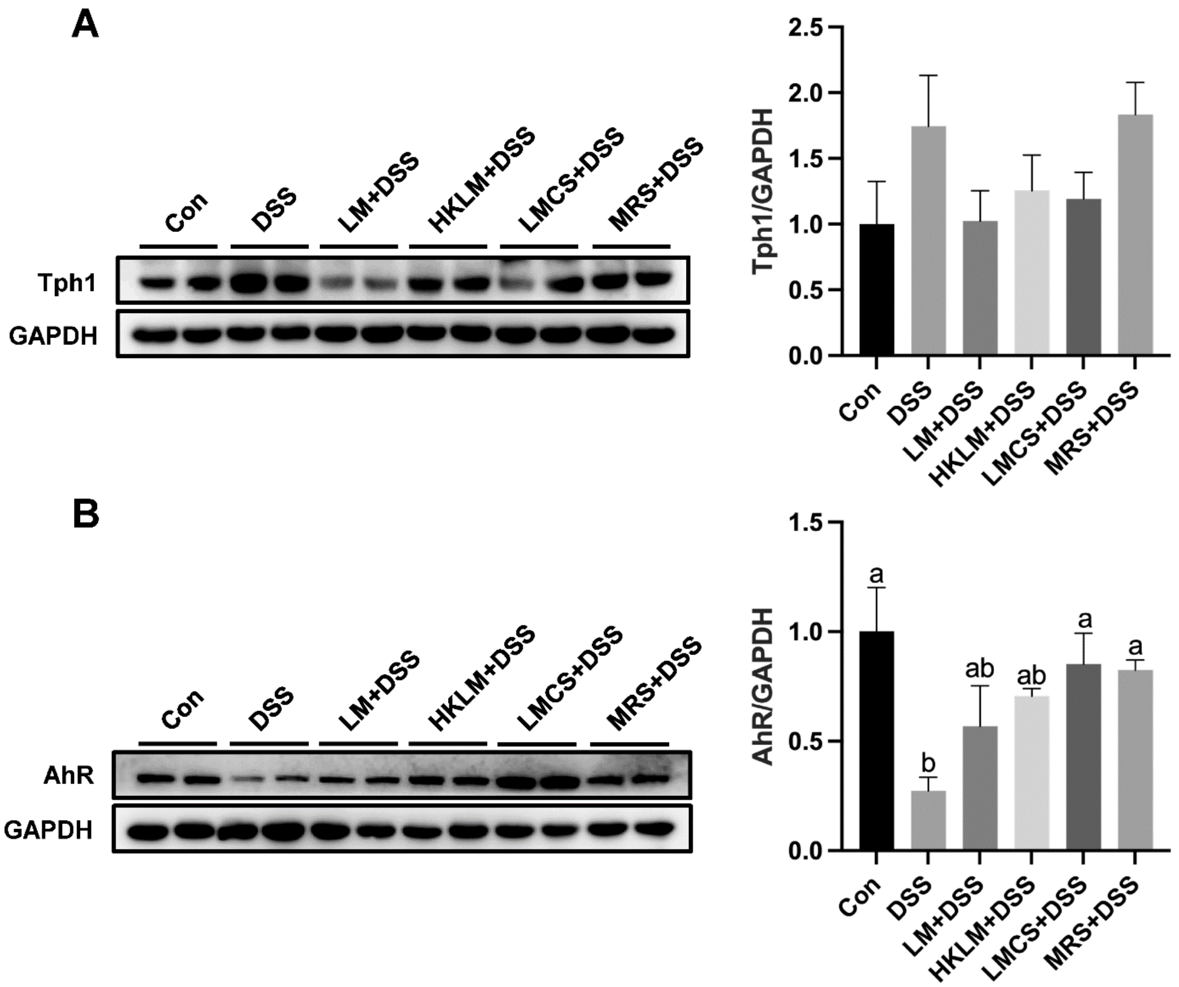

Supplemental Figure S1. Effects of different forms and components of LM treatment on the protein abundance of Tph1 (A) and AhR (B) in the colon of mice treated with DSS.

Values in the charts are means ± SEMs. a, b, values in the chart with different letters differ ($P < 0.05$).

AhR, Aryl hydrocarbon receptor; Con, control; DSS, dextran sodium sulfate; GAPDH, glyceraldehyde 3-phosphate dehydrogenase; HKLM, heat-killed *Limosilactobacillus mucosae*; LM, *Limosilactobacillus mucosae*; LMCS, *Limosilactobacillus mucosae* culture supernatant; MRS, de Man, Rogosa, and Sharp medium; Tph1, tryptophan hydroxylase 1.
